# Computational Study of the Binding Mechanism of Actin-Depolymerizing Factor 1 with Actin in *Arabidopsis thaliana*

**DOI:** 10.1371/journal.pone.0159053

**Published:** 2016-07-14

**Authors:** Juan Du, Xue Wang, Chun-Hai Dong, Jian Ming Yang, Xiao Jun Yao

**Affiliations:** 1 Key Laboratory of Plant Biotechnology of Shandong Province, College of Life Science, Qingdao Agricultural University, Qingdao, China; 2 College of Chemistry and Chemical Engineering, Lanzhou University, Lanzhou, China; Helmholtz Centre for Infection Research, GERMANY

## Abstract

Actin is a highly conserved protein. It plays important roles in cellular function and exists either in the monomeric (G-actin) or polymeric form (F-actin). Members of the actin-depolymerizing factor (ADF)/cofilin protein family bind to both G-actin and F-actin and play vital roles in actin dynamics by manipulating the rates of filament polymerization and depolymerization. It has been reported that the S6D and R98A/K100A mutants of actin-depolymerizing factor 1 (ADF1) in *Arabidopsis thaliana* decreased the binding affinity of ADF for the actin monomer. To investigate the binding mechanism and dynamic behavior of the ADF1–actin complex, we constructed a homology model of the AtADF1–actin complex based on the crystal structure of AtADF1 and the twinfilin C-terminal ADF-H domain in a complex with a mouse actin monomer. The model was then refined for subsequent molecular dynamics simulations. Increased binding energy of the mutated system was observed using the Molecular Mechanics Generalized Born Surface Area and Poisson–Boltzmann Surface Area (MM-GB/PBSA) methods. To determine the residues that make decisive contributions to the ADF1 actin-binding affinity, per-residue decomposition and computational alanine scanning analyses were performed, which provided more detailed information on the binding mechanism. Root-mean-square fluctuation and principal component analyses confirmed that the S6D and R98A/K100A mutants induced an increased conformational flexibility. The comprehensive molecular insight gained from this study is of great importance for understanding the binding mechanism of ADF1 and G-actin.

## Introduction

Actin is a highly conserved protein. As one of the most abundant proteins in most eukaryotic cells, actin plays important roles in cellular functions such as endocytosis, organelle movement, cell division, cell mobility, and maintenance of cell shape [1–3]. Those functions are influenced by rapid transitions between monomeric (G-actin) and filamentous (F-actin) states regulated by a large number of actin-binding proteins (ABPs) in the cell, such as capping proteins and severing proteins. Actin-capping proteins bind to actin and enhance filament depolymerization, and actin-severing proteins enhance fragmentation. Actin-depolymerizing factor (ADF)/cofilin proteins are actin-severing proteins, and are highly conserved among eukaryotes [4–8]. They bind to both monomeric and filamentous actin, and play vital and complicated roles in actin dynamics by controlling the rate of filament polymerization and depolymerization [9]. The ADF/cofilin proteins are involved in primary filament depolymerization, and facilitate actin turnover by severing actin filaments and increasing the rate of dissociation of actin monomers from the pointed ends of actin filaments [8, 10–12]. The activity of ADF/cofilin proteins is tightly controlled in response to various cellular activities. In plants, the activity of ADF is regulated by several factors such as N-terminal phosphorylation and pH [13–16].

In the genus *Arabidopsis*, there are 11 expressed members of the ADF family that are grouped into four ancient subclasses [17]. AtADF1 belongs to subclass I, and is strongly expressed in an extensive range of tissues including flowers, seedlings, roots, and mature leaves [10, 17, 18]. In 2000, the crystal structure of ADF1 from *Arabidopsis thaliana* was determined by Bowman et al.; it was the first ADF/cofilin structure from the plant kingdom to be determined [19]. However, the structure of an ADF in complex with actin was not determined until 2008 when Paavilainen et al. reported the crystal structure of the twinfilin C-terminal ADF-H domain in a complex with a mouse actin monomer (PDB ID: 3DAW), which indicated that the ADF-H domain binds to G-actin with the long α-helix inserted into the hydrophobic cleft between subdomains 1 and 3 of actin [20]. By then, numerous crystal structures of ADF/coffin from different organisms had been determined [21–28].

Dong et al. (2013) reported that in *A*. *thaliana* ADF1 is predominantly phosphorylated by AtCDPK6 at serine 6, which prevents ADF1 from binding to actin. The subsequent mutation experiment demonstrated that the S6D and R98A/K100A mutants of ADF1 in *A*. *thaliana* decreased the binding affinity of the ADF for both actin monomers and filaments [29, 30]. Others have explored the mechanism of interaction between ADF/cofilin and actin using computational methods. Wriggers et al. built a structure model of an ADF/cofilin-G-actin complex based on the crystal structure of the actin-gelsolin segment-1 complex by docking and molecular dynamics (MD) simulations [31–36]. Sept et al. then studied the association rate of actin monomers bound with ADF based on this model using the Brownian dynamics method [32]. The molecular interaction mechanism between cofilin and actin filaments has also been investigated using all-atom MD simulations, coarse-grained MD simulations, and normal mode analysis, which provided insight into the overall mechanism how ADF/cofilin binding influences the structure and mechanical properties of actin filaments [33–36]. However, detailed understanding of the direct molecular interactions between ADF and G-actin, and the dynamic behavior after ADF1 mutation in *A*. *thaliana*, were still lacking.

Here, we modeled an AtADF1 actin monomer complex structure based on the crystal structure of AtADF1 and the twinfilin C-terminal ADF-H domain in a complex with an actin monomer. We then refined the features of the model in order to perform MD simulations. Molecular Mechanics Generalized Born Surface Area and Poisson–Boltzmann Surface Area (MM-GB/PBSA) methods were used to calculate the binding free energy between ADF1 and actin. Per-residue decomposition and computational alanine scanning analyses were performed to determine the residues that make decisive contributions to ADF1 and actin binding affinity. Root-mean-square fluctuations (RMSFs) and principal component analysis confirmed that S6D and R98A/K100A mutations increased conformational flexibility. Our study provides a comprehensive molecular insight into the binding mechanism of ADF1 and G-actin, and gives an explanation for the reduced binding affinity of mutated ADF1 to G-actin at atomic level. Moreover, such information provides novel clues for further mutation experiments on the ADF/cofilin family.

## Materials and Methods

### Construction of simulation systems

The ADF–actin complex was built using *A*. *thaliana* ADF1 and actin1, based on the twinfilin C-terminal ADF-H domain in a complex with a mouse actin monomer (PDB ID: 3DAW). The crystal structure of AtADF1 (PDB ID: 1F7S) was obtained from the Research Collaboratory for Structural Bioinformatics (RCSB) Protein Data Bank [37] and aligned to the twinfilin C-terminal ADF-H domain. The missing residues (2-ANA-4, 129-MDLDVFRSRAN-139) were also built based on this structure by Modeller [38]. The sequence of actin1 (accession number: AEC09427.1) was downloaded from the National Center for Biotechnology Information (NCBI) [39]. A homology model of actin1 was built using the actin monomer of 3DAW as a template (protein sequence identity = 88%) by Modeller [38].

### Molecular dynamics simulation

The model was then refined to eliminate bad contacts by using Amber 14. The force field parameters for Mg^2+^ ion and ATP were downloaded from the Amber parameter database [40, 41]. A standard AMBER ff03.r1 force field was assigned to the protein [42, 43]. In the experimental work, the purification for ADF1 and actin were performed under different pH conditions (pH = 7 for ADF1 and pH = 8 for actin) [29]. The ionizable residues were predicted with the same protonation states under both conditions based on the pKa values calculated by the H++ server [44] and PROPKA3.1 [45, 46], which were consistent with the default set of AMBER. The protonation state of ionizable residues was set at the default value for pH 7. Systems were neutralized by adding Na^+^ ions. The built complex was solvated in a rectangular box filled with TIP3P water molecules [47], maintaining a 10-Å distance between any solute atom and the boundary. In the first stage, the water molecules and ions were relaxed by restraining the whole protein and ligands (5000 cycles of steepest descent and 2000 cycles of conjugate gradient minimizations); second, the side chains of the protein were relaxed by restraining the backbone of the proteins and ligands (5000 cycles of steepest descent and 2000 cycles of conjugate gradient minimizations); third, the whole system was relaxed without any restrains (5000 cycles of steepest descent and 5000 cycles of conjugate gradient minimizations). After minimization, the model was gradually heated from 0 to 300 K within 50 ps with the backbone of proteins restrained (500 kcal/mol/Å^2^) in the NVT ensemble. Then, the model was relaxed within 2.55 ns from 500 to 0 kcal/mol/Å^2^ in the NPT ensemble. The final equilibration phase lasted 1 ns without restraints.

All molecular dynamics simulations were performed using the GPU version of the PMEMD engine provided with Amber 14. First, the initial model was subjected to a 40 ns MD simulation to reach equilibrium. A snapshot was then extracted from the equilibrium stage as the wild type (WT) and the mutation was carried out. Four systems, ADF1-WT, ADF1-S6D, phosphorylated form ADF1-S6^phos^ and ADF1-R98A/K100A in a complex with actin, were subjected to simulations. The detailed protocol was mentioned above. MD simulations were conducted in the WT, ADF1-S6D, ADF1-S6^phos^ and ADF1-R98A/K10A systems at 100 ns to produce trajectories, respectively.

The covalent bonds to hydrogen atoms were constrained using the SHAKE algorithm, and the Particle Mesh Ewald (PME) method [48] was employed to calculate long-range electrostatic interactions. The cut-off for van der Waals interactions was set to 10 Å. The time step used for the simulations was set to 2 fs. The atom coordinates were saved every 10 ps for subsequent analysis.

### Binding free energy calculation

The MM-GB/PBSA methods were applied to calculate the binding free energies between ADF1 and actin [49–51]. For each system, 500 snapshots were collected from the 10 ns of the trajectory after equilibration with 20 ps intervals. The binding free energy between ADF1 (receptor) and actin (ligand) was calculated as follows:

$$\Delta G_{\text{binding}}=G_{\text{complex}}-G_{\text{receptor}}-G_{\text{lig}}{}_{\text{and}}=\Delta G_{\text{gas}}+\Delta G_{\text{solv}}$$ (1)

$$\Delta G_{\text{gas}}=\Delta H_{\text{gas}}-T\Delta S\approx \Delta E_{\text{MM}}-T\Delta S$$ (2)

$$\Delta G_{\text{binding}}\approx \Delta E_{\text{MM}}+\Delta G_{\text{solv}}-T\Delta S$$ (3)

$$\Delta E_{\text{MM}}=\Delta E_{\mathrm{int}}+\Delta E_{\text{vdw}}+\Delta E_{\text{ele}}$$ (4)

$$\Delta G_{\text{solv}}=\Delta G_{\text{GB/PB}}+\Delta G_{\text{SA}}$$ (5)

$$\Delta G_{\text{SA}}=\gamma \;*\;\Delta \text{A}+\beta$$ (6)

$$SE=\frac{STD}{\sqrt{N}}$$ (7)

*G*_complex_, *G*_receptor_, *G*_ligand_ are the free energies of complex, receptor, and ligand, respectively. According to MM-PBSA and MM-GBSA theory, the binding free energy (Δ*G*_binding_) is composed of two parts: the gas phase molecular mechanical (MM) energy (Δ*G*_gas_) and the solvation free energy (Δ*G*_solv_). It can also be decomposed into three terms: the molecular mechanical energy term (Δ*E*_MM_, a sum of the changes of Δ*E*_int_, Δ*E*_ele_, and Δ*E*_vdw_), the solvation energy term (Δ*G*_solv_), and the vibrational entropy term (*T*Δ*S*). Δ*E*_int_, Δ*E*_ele_, and Δ*E*_vdw_ are given as internal energy contribution, electrostatic, and van der Waals interaction terms, respectively. Δ*E*_int_ is canceled between ligand, receptor, and complex by using a single trajectory strategy, and it can significantly reduce the noise in most cases. The change of solvation energy (Δ*G*_solv_) comprises the polar component (Δ*G*_GB/PB_) and the nonpolar component of the desolvation energy (Δ*G*_SA_). The polar solvation contribution (Δ*G*_GB/PB_) can be calculated using the Poisson–Boltzmann (PB) and Generalized Born (GB) equation. The dielectric constant for solvent was set to 80 and for solute was set to 1, 2 or 4, respectively. The nonpolar component of the desolvation energy (Δ*G*_SA_) can be estimated using Eq 6, where ΔA represents the change of the solvent-accessible surface area (SASA) of the system calculated using the LCPO algorithm [52], and the fitting coefficients γ and β were set to 0.0072 kcal/mol·Å^2^ and 0 in GB [53], and 0.00542 kcal/mol·Å^2^ and 0.92 kcal/mol in PB, respectively [53–55]. The term (*T*Δ*S*) in Eq 2 is the change in the conformational entropy upon ligand binding. Here, normal-mode analysis (NMA) was used for the calculation of the conformational entropy [56], which was calculated from the sum of translational, rotational, and vibrational components, and as there was high computational demand, only 125 snapshots extracted from the 10 ns of the MD trajectories after equilibration with 80 ps intervals were used. All the atoms in ADF1-actin complex were used for the normal mode calculation. Each snapshots were subjected to energy minimization for 10000 steps in the presence of a distance-dependent dielectric of 4*r*(*i*,*j*) (where *r*(*i*,*j*) is the distance between two atoms) until the root-mean-square of the elements of the energy gradient vector is less than 0.001 kcal mol^-1^ Å^-1^. The mass-weighted Hessian matrix for each minimized snapshot was calculated and diagonalized by using normal mode analysis. The obtained frequency of the normal mode was used to calculate the entropy.

The calculation error bars are standard errors (*SE*) calculated using Eq 7; the *STD* is the standard deviation and *N* is the number of trajectory snapshots used in the calculation.

To further investigate the molecular determinants of ADF1 binding to actin, the effective binding energies calculated using the MM-PBSA method were decomposed into the contributions from individual residues [57].

### Computational alanine scanning

The computational alanine scanning method involves replacing the side chain of a given residue (except glycine or proline) with a methyl group (alanine), then recalculating the absolute binding free energy of the mutated system [58, 59]. In this work, the binding free energy of the alanine mutant was calculated using the MM-PBSA approach described above, with the snapshots for the wild type complex. All energy terms were calculated for 500 snapshots along the last 10 ns trajectory with 20 ps intervals. ΔΔ*G*_*bind*_ was defined by the following equation, where Δ*G*_*bind*_ is the summation of the molecular mechanical energy term (Δ*E*_MM_) and the solvation energy term (Δ*G*_solv_).

$$\Delta \Delta G_{bind}=\Delta G_{bind}^{\text{mutant}}-\Delta G_{bind}^{\text{wildtype}}$$

### Principal component analysis

Principal component analysis was carried out using the PTRAJ module of AmberTools. Five thousand snapshots were taken from the MD simulation trajectories. To obtain the proper trajectory matrix, overall translation or rotation motion were removed by fitting the coordinate data to the average structure. The trajectory data were then utilized to generate a covariance matrix between the Cα atoms *i* and *j*, defined as:

$$C_{ij}=<(x_{i}-<x_{i}>)(x_{j}-<x_{j}>)>(i,j=1,2,3,\ldots ,3N)$$

Where *x*_*i*_ and *x*_*j*_ are Cartesian coordinates of the *i*th and *j*th Cα atom, *N* is the number of the Cα atoms considered, < *x*_*i*_ > and < *x*_*j*_ > represent the time average over all the configurations obtained in the MD simulation [60–62].

## Results and Discussion

### Dynamics behavior of the WT and mutant systems

**Fig 1:**
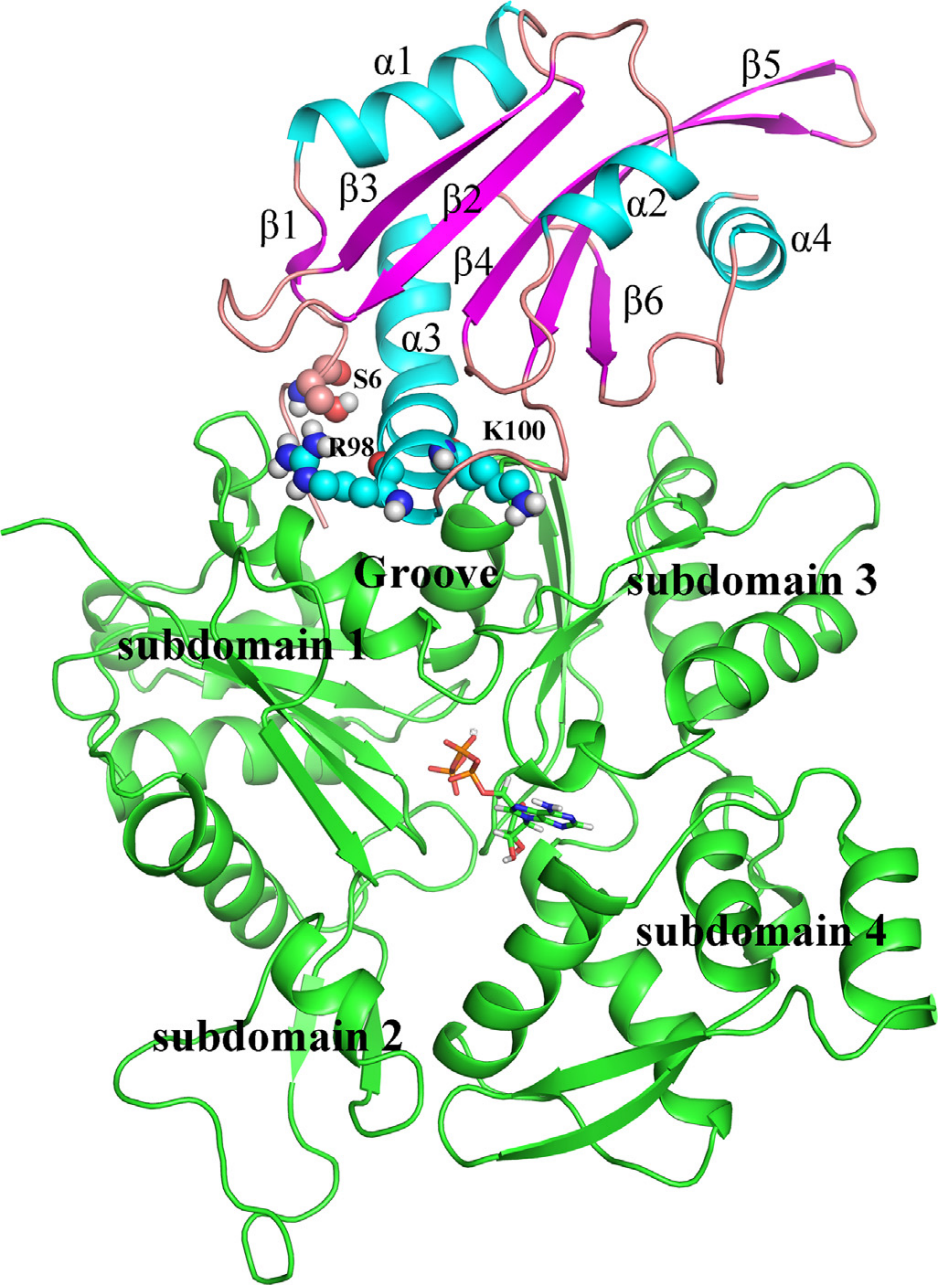
The overall structure of actin-depolymerizing factor 1 (ADF1) in complex with actin1 monomer. ADF1 is depicted with colored secondary structure (cyan for α-helix, magenta for β-sheet, and salmon for random coil). Actin is colored green. The mutated residues are shown as spheres.

ADF1 is composed of four α-helices and six β-strands (Fig 1). ADF1 binds to actin with the long α-helix inserted into the hydrophobic cleft (groove) between subdomains 1 and 3 of the actin. The constructed complex was subjected to a 40-ns MD simulation to reach equilibrium. Subsequently, a snapshot was extracted from the equilibrium stage as the WT, and used as a starting point for the mutations. The protein stability of the four systems (ADF1-WT, ADF1-S6D, ADF1-S6^phos^ and ADF1-R98A/K100A in complex with actin) during the MD simulations was monitored by root mean square deviation (RMSD) of the backbone atoms (S1 Fig). The WT system reached equilibrium after 60 ns with average RMSDs of 1.80 Å. The ADF1-S6D and ADF1-S6^phos^ systems reached equilibrium after 30 ns with average RMSDs of 1.80 Å and 1.96 Å, respectively. However, the ADF1-R98A/K100A system stabilized after 70 ns with a larger average RMSD value of 2.56 Å. Thus, based on the RMSD results, our MD simulations are reliable enough for further investigation.

**Fig 2:**
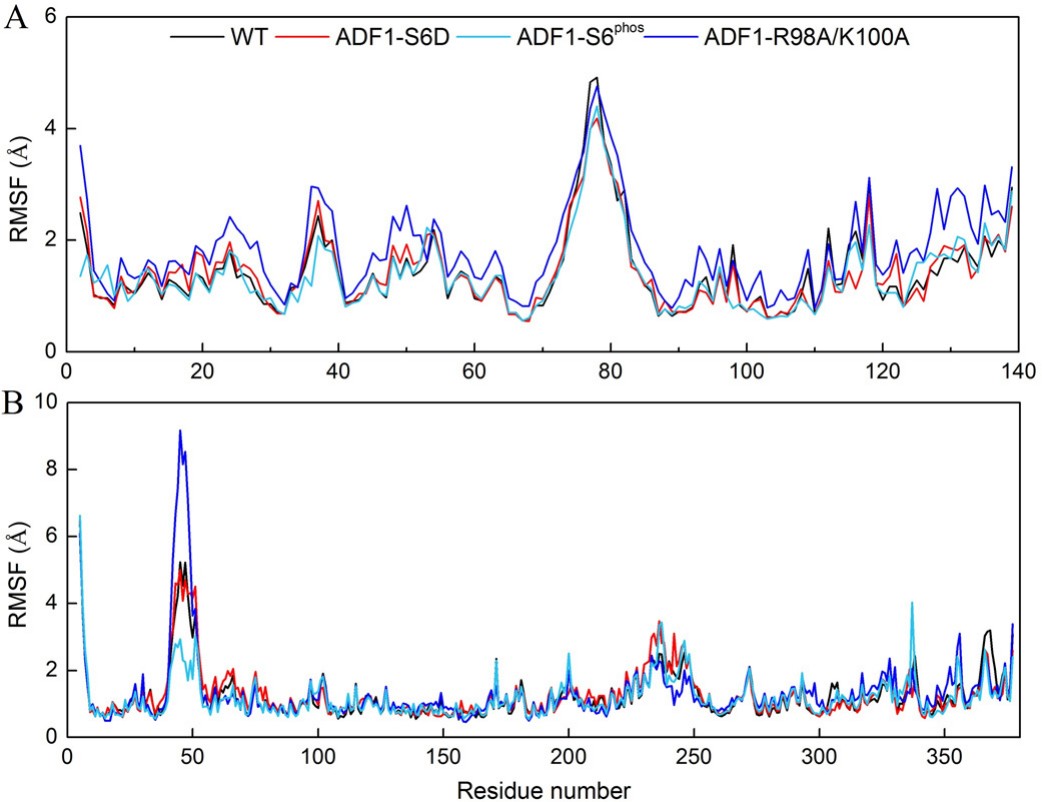
Root-mean-square fluctuation (RMSF) plots of Cα atoms for wild type (WT) (black), ADF1-S6D (red), ADF1-S6^phos^ (green cyan) and ADF1-R98A/K100A (blue) during the molecular dynamics (MD) simulations. (A) RMSF of ADF1; (B) RMSF of actin.

To identify which parts of the complex contributed most to protein mobility, the RMSF of the four systems versus the residue number was investigated (Fig 2). Three major sites were distinguished in ADF1 that interact with actin: the N-terminal loop, the α3-helix, and the β6–α4 loop [43]. It suggests that the R98A/K100A mutation in ADF1 induces a larger fluctuation on almost the whole ADF1 protein compared with the WT, especially in the N-terminal loop (residues 2–8) and the β6–α4 loop (residues 116–139) (Fig 2A). The protein mobility of ADF1 in the S6D mutation is slightly larger than in the WT. The residues with higher flexibility include residues 16–29 (belonging to the α1 helix) and Gln122 (belonging to the β6-strand). The protein mobility of ADF1 with phosphorylated Ser6 varies not significant from the WT, compared with the mutated systems. The flexibilities of the actins in the three systems are similar except for residues 36–50, which constitute a long loop belonging to subdomain 2 that is far away from the binding interface. Residues 349–354, which interact with the N-terminal loop of ADF1, became more flexible after ADF1-R98A/K100A mutation (Fig 2B).

**Fig 3:**
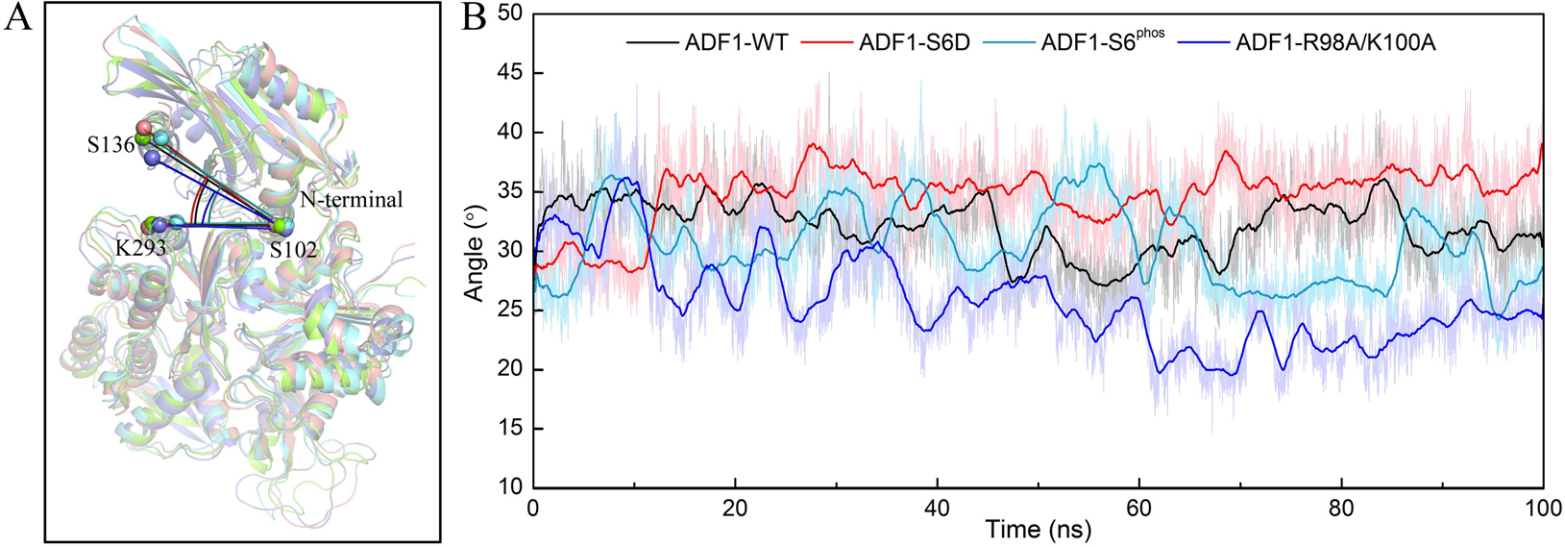
The rigid rotation of actin-depolymerizing factor 1 (ADF1) relative to actin. (A) The representative structure from the last 30 ns of the molecular dynamics (MD) trajectories of the mutation system (ADF1-S6D, salmon; ADF1-S6^phos^, green cyan; ADF1-R98A/K100A, marine) superimposed on the wild type (WT) (green) by the secondary structure of actin; (B) Time-dependent rigid rotation angles of ADF1-WT (black), ADF1-S6D (red), ADF1-S6^phos^ (green cyan), and ADF1-R98A/K100A (blue) systems.

As can be seen from the RMSD and RMSF results, ADF1 was more flexible after mutation. To further explore the dynamic behavior of the complex, representative structures from the last 30 ns of the MD simulation trajectories of the mutation systems were superimposed on the WT according to the secondary structure of actin (Fig 3A). A noticeable rigid rotation of ADF1 versus actin was observed, which brought the N-terminal loop in ADF1 away from the actin subdomain 1, and brought the α4-helix in ADF1 close to the actin subdomain 3 in the ADF1-R98A/K100A system. The angle, represented by the Cα atoms of the three residues (Lys293 in actin, Ser102 and Ser136 in ADF1) was measured to represent the degree of this rotation (Fig 3A). The rotation angles of the four systems were monitored during the MD simulation (Fig 3B), which shows that the rotation angle in the WT system was larger than in the ADF1-R98A/K100A system and smaller than that in ADF1-S6D system. The averaged rotation angle during the equilibrium stage of the WT, ADF1-S6D, ADF1-S6^phos^ and ADF1-R98A/K100A systems were 31.74±2.58°, 35.25±2.23°, 29.62±2.96° and 23.21±2.19°, respectively.

### Total binding free energy and per-residue contributions between ADF1 and actin predicted by MM-GB/PBSA methods

Generally, a low dielectric constant of ε = 1 is used for solute in Molecular Mechanics Generalized Born Surface Area and Poisson–Boltzmann Surface Area (MM-GB/PBSA) methods [63]. Larger values ε = 2 or ε = 4 are also reported [50, 64].

We compared the binding free energies calculated using three different solute dielectric constants, 1, 2 and 4. The results were listed in S1 Table. The predicted binding free energy for the WT system is always smaller than the ADF1-R98A/K100A system under three settings. The predicted binding free energy for the WT system is smaller than the ADF1-S6D system with the setting of ε = 1. When ε = 2 and 4, the predicted binding free energy for the WT system is larger than the ADF1-S6D system, which is not consistent with the conclusion in the experimental work [29]. The optimal value of the protein dielectric constant is still an problem need to be discussed in the literature [65]. In this work, ε = 1 made the best predictions. Based on the best results, the predicted binding free energy with the setting of ε = 1 is summarized in Table 1.

As shown in Table 1, both the MM-GBSA and MM-PBSA results suggest that the phosphorylation of Ser6, S6D and R98A/K100A mutations of ADF1 led to reduced binding affinity. The predicted results are in agreement with the experimental findings that after mutation the binding affinity of ADF1 and actin decreases [29, 30]. Further analysis suggested that the main differences in binding free energy between the WT and the mutations arose from van der Waals interactions and the electrostatic energy (sum of the electrostatic solvation free energy and MM electrostatic energy, Δ*G*_GB_/Δ*G*_PB_ + Δ*E*_ele_) contribution. The calculated van der Waals contributions in the WT, ADF1-S6D, ADF1-S6^phos^ and ADF1-R98A/K100A systems were -107.15 kcal/mol, -111.78 kcal/mol, -113.34 kcal/mol and -107.49 kcal/mol, respectively. The electrostatic energy values for the WT, ADF1-S6D, ADF1-S6^phos^ and ADF1-R98A/K100A systems were 32.19/47.96 kcal/mol, 38.55/56.07 kcal/mol, 36.69/59.76 kcal/mol, and 36.09/57.01 kcal/mol in MM-GB/PBSA, respectively.

**Table 1:** Binding free energy for the four systems according to the MM-GB/PBSA methods.

Method				MM-GB/SA			MM-PB/SA		
System	Δ*E*_ele_	Δ*E*_vdw_	-*T*Δ*S*	Δ*G*_SA_	Δ*G*_GB_	Δ*G*_binding_^a^	Δ*G*_SA_	Δ*G*_PB_	Δ*G*_binding_^a^
**WT**	-572.37±2.70	-107.15±0.24	47.44±0.74	-14.29±0.03	604.56±2.56	-41.82	-13.81±0.03	620.33±2.59	-25.56
**S6D**	-583.14±2.40	-111.78±0.27	51.31±0.71	-15.45±0.04	621.69±2.22	-37.37	-14.90±0.02	639.21±2.33	-19.30
**S6**^**phos**^	-369.87±3.68	-113.34±0.23	50.80±0.71	-15.70±0.04	406.56±3.49	-41.56	-14.48±0.03	429.63±3.70	-17.25
**R98A/K100A**	-465.62±2.45	-107.49±0.29	49.00±0.78	-14.98±0.04	501.71±2.31	-37.38	-14.98±0.04	522.63±2.42	-16.46

^a^All energies are in kcal/mol.

**Fig 4:**
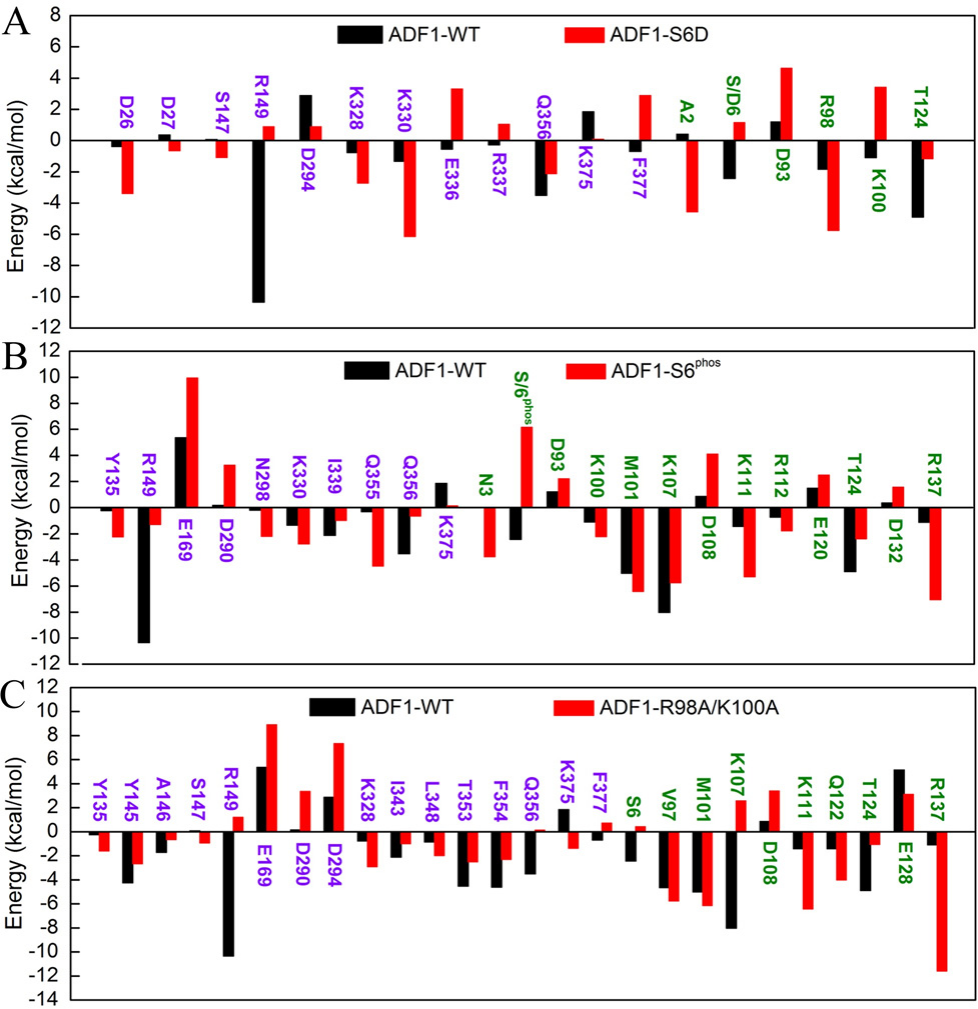
Per-residue energy contribution plots. The residues displayed as purple belong to actin1 and the green residues belong to ADF1. (A). Comparison between wild type (WT) and ADF1-S6D systems of per-residue energy contributions; (B). Comparison between wild type (WT) and ADF1-S6^phos^ systems of per-residue energy contributions; (C). Comparison between WT and ADF1-R98A/K100A systems of per-residue energy contributions.

Therefore, to achieve a more precise quantitative interpretation of binding affinity, per-residue basis binding free energy decomposition was performed to determine the individual energy contribution to the interaction energy (S2 Table). A residue was reported only if the per-residue energy contribution difference between the WT and the mutation systems was larger than 1.00 kcal/mol (Fig 4). The ADF1 S6D mutation affected binding affinity mainly through residues Asp26, Asp27, Ser147, Arg149, Asp294, Lys328, Lys330, Arg337, Gln356, Lys375 and Phe377 in actin, and residues Ala2, Ser/Asp6, Asp93, Arg98, Lys100 and Thr124 in ADF1 (Fig 4A). The summation of the energy contribution of Glu128 (ADF1) and Arg149 (actin) is -5.19 kcal/mol in the WT system versus 5.35 kcal/mol in the ADF1-S6D system (S2 Table). These two residues form hydrogen bond interactions in the WT system. The summation of the energy contribution of Ser6/Asp6 (ADF1) and Phe354 (actin) is -7.03 kcal/mol in the WT system versus -3.02 kcal/mol in the ADF1-S6D system (S2 Table). These two residues form OH-π interactions in the WT system. To calculate the OH-π interaction strength, 5000 snapshots were extracted to measure the angle of OH with the center of the benzene and the distance between the H atom of OH and the center of the benzene. Only an angle between 65° and 90° and a distance between 2.0 Å and 3.5 Å indicated a strong interaction. We observed that the hydroxyl of Ser6 in ADF1 formed a stable OH-π interaction with the benzene ring of Phe354 in actin in most of the MD trajectories in the WT system (S2A Fig). In the S6D mutation, the hydroxyl group (-OH) was substituted with a carboxy group (-COOH). The electrostatic energy contributions of Phe354 and Ser/Asp6 were 0.13 kcal/mol (WT) and 4.75 kcal/mol (ADF1-S6D), which means that a charged residue is unfavorable for the binding affinity. After S6D mutation, most of the interaction residues became unfavorable to the binding affinity except Asp26, Lys328 and Lys330 in actin, and Ala2 and Arg98 in ADF1.

The phosphorylation of Ser 6 affected binding affinity mainly through many residues (Fig 4B). The significant changes included the following interactions between ADF1 and actin. The summation of the energy contribution of Glu128 (ADF1) and Arg149 (actin) is -5.19 kcal/mol in the WT system versus 2.94 kcal/mol in the ADF1-S6^phos^ system (S2 Table). The summation of the energy contribution of Ser6 (ADF1) and Phe354 (actin) is -7.03 kcal/mol in the WT system versus 1.85 kcal/mol in the ADF1-S6^phos^ system (S2 Table). The electrostatic energy contributions of Phe354 and Ser6 were 0.13 kcal/mol (WT) and 9.34 kcal/mol (ADF1-S6^phos^).

We can see that with the R98A/K100A mutation, more residues changed the energy contribution (Fig 4C). The significant change was the increase of the summation of the energy contribution of Glu128 (ADF1) and Arg149 (actin), which is -5.19 kcal/mol in the WT system versus 4.36 kcal/mol in the ADF1- R98A/K100A system (S2 Table). Another significant change was the increase of the energy contribution of Ser6 (belonging to the ADF1 N-terminal loop), and T353, F354, and Q356 (belonging to actin subdomain 1). The significant reduction in the energy contribution was caused by the rigid rotation of ADF1, which brings the N-terminal loop away from the interaction surface and breaks the interactions between the residues mentioned above. In particular, the OH-π interaction between Ser6 in ADF1 and Phe354 in actin disappeared in most of the trajectories in the ADF1-R98A/K100A system (S2B Fig). The rigid rotation of ADF1 brings the α4-helix, the β6-strand, and some residues in the α3-helix close to subdomain 3 in actin, forming new interactions, including residues Gln122 (belonging to the β6-strand) and Arg137 (belonging to the α4-helix) and residues Asp290 and Asp294 in actin subdomain 3, and residues Lys107 and Lys111 (belonging to the α4-helix) and residue Glu169 in actin subdomain 3. The summation of the energy contribution of these residues changed considerably, which is also unfavorable for the binding of the ADF–actin complex. The overall energy contributions from the residues mentioned above were -3.56 kcal/mol (WT system) and 0.18 kcal/mol (R98A/K100A mutation system) (S2 Table).

### Computational alanine scanning on ADF1 residues

**Fig 5:**
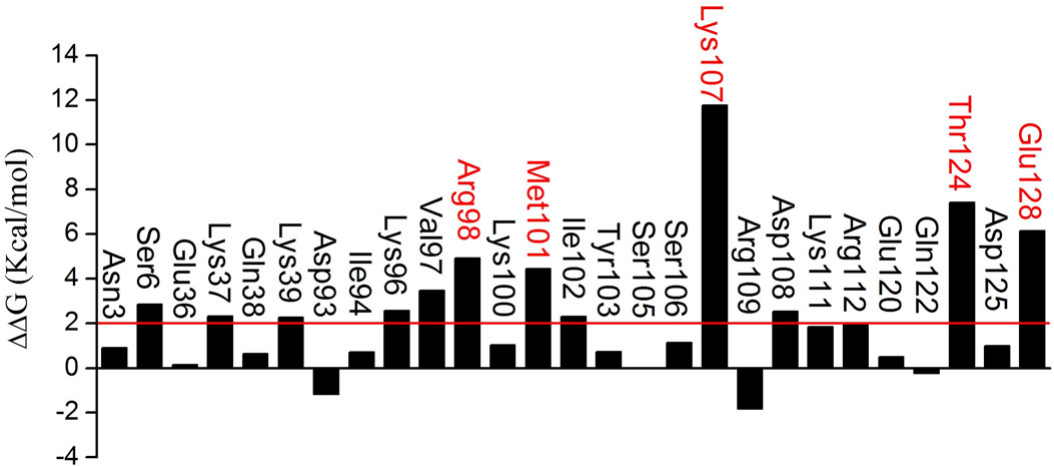
ΔΔ*G*_*bind*_ for each mutated residue of actin-depolymerizing factor 1 (ADF1) obtained from computational alanine scanning.

Residues at 8 Å around the binding surface in ADF1 were calculated except for glycine and proline. The results of computational alanine scanning for ADF1 residues are shown in Fig 5. A positive ΔΔ*G_bind_* value means that the residue was energetically more favorable than the corresponding alanine residue. The residues with a ΔΔ*G_bind_* value larger than 2.00 kcal/mol were considered hot spots. As can be seen, there are twelve “hot spot” residues on ADF, which belong to the α3-helix (residues 97–111), namely Lys96, Val97, Arg98, Met101, Ile102, Lys107, and Lys111. The top five hot spots, with ΔΔ*G_bind_* larger than 4.00 kcal/mol, were Lys98, Met101, Lys107, Thr124 and Glu128. Some of the predicted hot spots, including Arg98, Met101, and Ile102, are supported by a previous site mutagenesis study [66]. The other residues, including Lys96, Val97, Lys107, Lys111, Thr124 and Glu128, which were not identified in the former study [66], are worthy of verification by further mutagenesis experiments.

### Larger magnitudes of the atomic fluctuations in the mutation systems than the WT system

**Fig 6:**
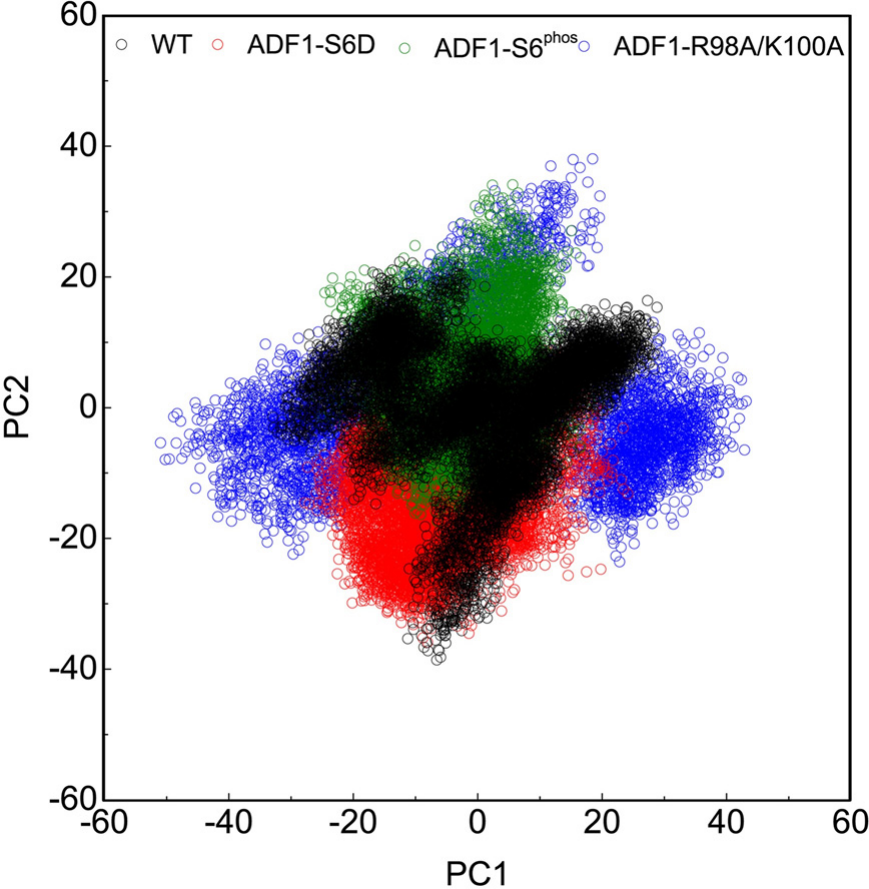
Principal component analysis (PCA) plot of wild type (WT) (black), ADF1-S6D (red), ADF1-S6^phos^ (green), and ADF1-R98A/K100A (blue).

**Fig 7:**
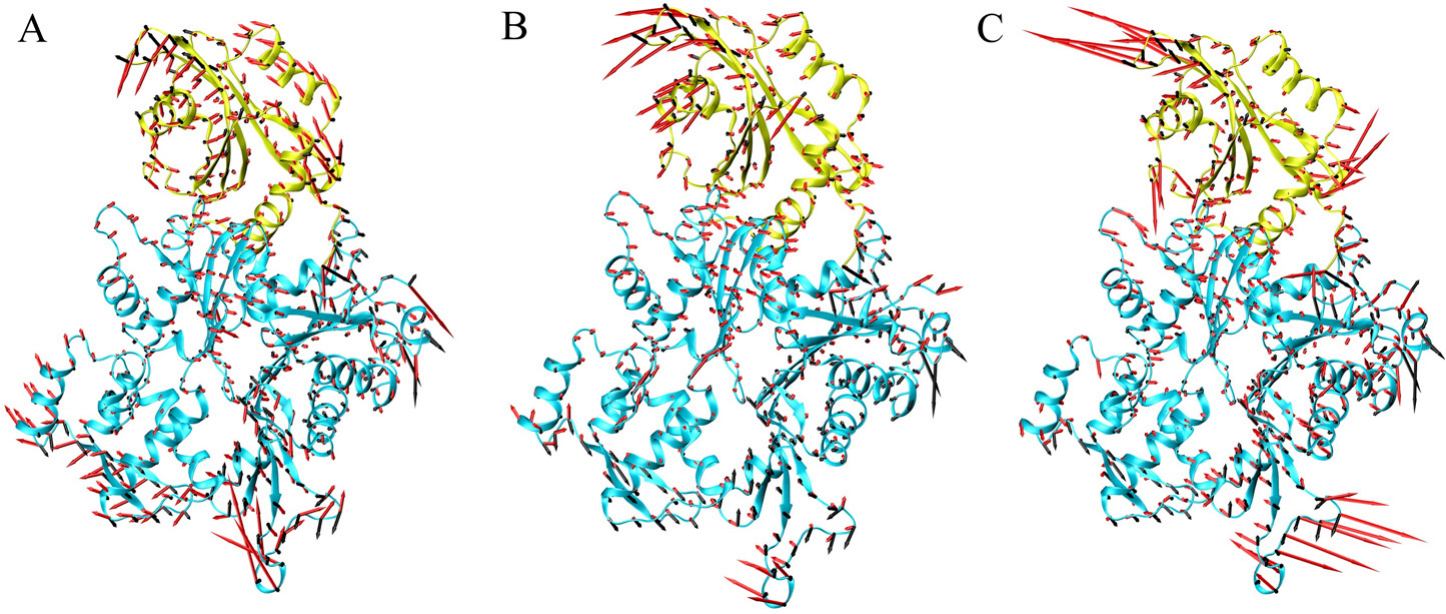
Porcupine plots of the eigenvectors. Actin and actin-depolymerizing factor 1 (ADF1) shown in cyan and yellow, respectively. The arrows attached to each backbone atom indicate the direction of the eigenvector and the size of each arrow shows the magnitude of the corresponding eigenvalue. (A). Comparison of wild type (WT) (black arrow) and ADF1-S6D (red arrow) systems; (B). Comparison of wild type (WT) (black arrow) and ADF1-S6^phos^ (red arrow) systems; (C). Comparison of WT (black arrow) and ADF1-R98A/K100A (red arrow) systems.

Principal component analysis identifies and quantifies which collective atomic motions contribute most to the overall motion of the molecule during simulation. PCA on the conformations of the WT, phosphorylated and mutation systems highlighted significant differences in the motion as a result of the mutations and phosphorylation. From the PCA plot, it is clear that eigenvectors computed from the MD trajectory for the WT/ADF1-S6D, WT/ADF1-S6^phos^ and WT/ADF1-R98A/K100A systems varied greatly, which clearly indicates the difference in protein motions as a result of mutations and phosphorylation (Fig 6). To get a direct observation of protein motion after equilibrium, we performed PCA and presented the results as porcupine plots. We can observe that the motions of the four systems were qualitatively different (Fig 7). The magnitudes of the atomic fluctuations in ADF1-S6D, ADF1-S6^phos^ and ADF1-R98A/K100A systems were larger than that in the WT system. The mutation systems also induced large atomic fluctuations in actin. The ADF1-S6D had a more complicated motion direction that resulted from contributions of the N-terminal loop, the α1-helix, and the β4–β5 loop (Fig 7A). The motion of ADF1-S6^phos^ varies from the WT and ADF1-S6D, which mainly resulted form the contributions of the α4-helix, and the β4–β5 loop (Fig 7B). It is clear that the motion of ADF1-R98A/K100A primarily resulted from contributions of the N-terminal loop, the β4–β5 loop, and the β6–β4 loop (Fig 7C).

## Conclusion

In the present study, the binding mechanism of ADF1 with actin1 was explored using a combined computational protocol. Based on the snapshots from the MD simulations, the binding free energy between WT, mutated and phophorylated ADF1 and actin was calculated by the MM-PB/GBSA method, which indicated that the binding between ADF1 and actin was tighter in the WT than in the mutated and phosphorylated forms (ADF1-S6D, ADF1-R98A/K100A and ADF1-S6^phos^). The van der Waals and electrostatic energy (the sum of the electrostatic solvation free energy and MM electrostatic energy) contribution differences were the main factors affecting the binding affinity. Further computational alanine scanning found critical residues, such as Arg98, Met101, Lys107, Thr124 and Glu128, and interactions that are important for ADF1 and actin binding affinity. The dynamic behavior studies using RMSF and PCA showed that ADF1-S6D and ADF1-R98A/K100A induced larger flexibility to the protein compared with the WT. Rigid rotation was triggered, which broke the interaction between the N-terminal loop and residues 353–356 in actin subdomain 1. In summary, the present study underlines the use of MD simulations in combination with MM-GB/PBSA free energy calculations to provide a detailed description of the binding mechanism of ADF1 and actin at the atomic level. Through per-residue decomposition and computational alanine scanning, a list of the important residues for ADF1 and actin binding were determined, which provides clues for further mutation experiments on the ADF/cofilin family.

## Supporting Information

S1 Fig Backbone RMSDs are shown for WT (A), ADF1-S6D (B), ADF1-S6^phos^ (C) and ADF1-R98A/K100A system (D), respectively.
(TIF)

S2 Fig Comparison of the OH-π interaction in WT and ADF1-R98A/K100A systems. Dots in red square indicate snapshots with strong OH-π interaction. (A). The OH-π interaction of WT system; (B). The OH-π interaction of ADF1-R98A/K100A system.
(TIF)

S1 Table Binding free energy for the WT and mutated systems according to the MM-GB/PBSA methods based on different protein dielectric constants (kcal/mol). (DOCX)

S2 Table The contributions of the important residues for the binding of ADF1 with actin (kcal/mol). (DOCX)

## References

[1] Dong C-H, Kost B, Xia G, Chua N-H. Molecular identification and characterization of the Arabidopsis AtADF1, AtADF5 and AtADF6 genes. Plant Molecular Biology. 2001;45:517–27. 10.1023/A:1010687911374 11414611

[2] Kandasamy MKM, Elizabeth C.Roy Eileen; Meagher Richard B. Ascomycete fungal actins differentially support plant spatial cell and organ developmen. Cytoskeleton. 2015;72(2):80–92. 10.1002/cm.21198 25428798

[3] Su H, Zhu J, Cai C, Pei W, Wang Jiaojiao, Dong H. FIMBRIN1 Is Involved in Lily Pollen Tube Growth by Stabilizing the Actin Fringe. The Plant Cell. 2012;24:4539–54. 10.1105/tpc.112.099358 23150633 PMC3531851

[4] Carlier M-F. Control of actin dynamics. Current Opinion in Cell Biology. 1998;10(1):45–51. 10.1016/S0955-0674(98)80085-9 9484594

[5] Sun T, Li S, Ren H. Profilin as a regulator of the membrane-actin cytoskeleton interface in plant cells. Frontiers in Plant Science. 2013;4(512):1–7. 10.3389/fpls.2013.00512 24391654 PMC3867660

[6] Maiti S, Bamburg JR. Actin-Capping and -Severing Proteins In: Lane WJLD, editor. Encyclopedia of Biological Chemistry. Waltham: Academic Press; 2013 p. 18–26.

[7] Moon A, Drubin DG. The ADF/Cofilin Proteins: Stimulus-responsive Modulators of Actin Dynamics. Molecular Biology of the Cell. 1995;6(11):1423–31. 10.1091/mbc.6.11.1423 8589446 PMC301301

[8] Bamburg JR. Proteins of the ADF/Cofilin Family: Essential Regulators of Actin Dynamics. Annual Review of Cell and Developmental Biology. 1999;15(1):185–230. 10.1146/annurev.cellbio.15.1.185 10611961

[9] Roland J, Berro J, Michelot A, Blanchoin L, Martiel J-L. Stochastic Severing of Actin Filaments by Actin Depolymerizing Factor/Cofilin Controls the Emergence of a Steady Dynamical Regime. Biophysical Journal. 2002;94(6):2082–94. 10.1529/biophysj.107.121988 PMC2257902 18065447

[10] Maciver S, Hussey P. The ADF/cofilin family: actin-remodeling proteins. Genome Biology. 2002;3(5):1–12. 10.1186/gb-2002-3-5-reviews3007 PMC139363 12049672

[11] Qu X, Zhang H, Xie Y, Wang J, Chen N, Huang S. Arabidopsis Villins Promote Actin Turnover at Pollen Tube Tips and Facilitate the Construction of Actin Collars. The Plant Cell. 2013;25(5):1803–17. 10.1105/tpc.113.110940 23715472 PMC3694707

[12] Carlier M-F, Laurent V, Santolini J, Melki R, Didry D, Xia G-X, et al Actin Depolymerizing Factor (ADF/Cofilin) Enhances the Rate of Filament Turnover: Implication in Actin-based Motility. The Journal of Cell Biology. 1997;136(6):1307–22. 10.1083/jcb.136.6.1307 9087445 PMC2132522

[13] Smertenko AP, Jiang C-J, Simmons NJ, Weeds AG, Davies DR, Hussey PJ. Ser6 in the maize actin-depolymerizing factor, ZmADF3, is phosphorylated by a calcium-stimulated protein kinase and is essential for the control of functional activity. The Plant Journal. 1998;14(2):187–93. 10.1046/j.1365-313X.1998.00107.x 9669865

[14] Allwood EG, Anthony RG, Smertenko AP, Reichelt S, Drobak BK, Doonan JH, et al Regulation of the Pollen-Specific Actin-Depolymerizing Factor LlADF1. The Plant Cell. 2002;14(11):2915–27. 12417710 10.1105/tpc.005363 PMC152736

[15] Bernstein BW, Bamburg JR. ADF/Cofilin: a functional node in cell biology. Trends in Cell Biology. 2010;20(4):187–95. 10.1016/j.tcb.2010.01.001 20133134 PMC2849908

[16] Allwood EG, Smertenko AP, Hussey PJ. Phosphorylation of plant actin-depolymerising factor by calmodulin-like domain protein kinase. FEBS Letters. 2001;499(1–2):97–100. 10.1016/S0014-5793(01)02528-5 11418120

[17] Ruzicka DR, Kandasamy MK, McKinney EC, Burgos-Rivera B, Meagher RB. The ancient subclasses of Arabidopsis ACTIN DEPOLYMERIZING FACTOR genes exhibit novel and differential expression. The Plant Journal. 2007;52(3):460–72. 10.1111/j.1365-313X.2007.03257.x 17877706

[18] Feng Y, Liu Q, Xue Q. Comparative study of rice and Arabidopsis Actin-depolymerizing factors gene families. Journal of Plant Physiology. 2006;163(1):69–79. 10.1016/j.jplph.2005.01.015 16360805

[19] Bowman GD, Nodelman IM, Hong Y, Chua N-H, Lindberg U, Schutt CE. A comparative structural analysis of the ADF/Cofilin family. Proteins: Structure, Function, and Bioinformatics. 2000;41(3):374–84. 10.1002/1097-0134(20001115)41:3<374::AID-PROT90>3.0.CO;2-F 11025548

[20] Paavilainen VO, Oksanen E, Goldman A, Lappalainen P. Structure of the actin-depolymerizing factor homology domain in complex with actin. The Journal of Cell Biology. 2008;182(1):51–9. 10.1083/jcb.200803100 18625842 PMC2447895

[21] Fedorov AA, Lappalainen P, Fedorov EV, Drubin DG, Almo SC. Structure determination of yeast cofilin. Nat Struct Mol Biol. 1997;4(5):366–9. 10.1038/nsb0597-366 9145106

[22] Pope BJ, Zierler-Gould KM, Kühne R, Weeds AG, Ball LJ. Solution Structure of Human Cofilin: ACTIN BINDING, pH SENSITIVITY, AND RELATIONSHIP TO ACTIN-DEPOLYMERIZING FACTOR. Journal of Biological Chemistry. 2004;279(6):4840–8. 10.1074/jbc.M310148200 14627701

[23] Hellman M, Paavilainen VO, Naumanen P, Lappalainen P, Annila A, Permi P. Solution structure of coactosin reveals structural homology to ADF/cofilin family proteins. FEBS Letters. 2004;576(1):91–6. 10.1016/j.febslet.2004.08.068 15474017

[24] Goroncy AK, Koshiba S, Tochio N, Tomizawa T, Sato M, Inoue M, et al NMR solution structures of actin depolymerizing factor homology domains. Protein Science. 2009;18(11):2384–92. 10.1002/pro.248 19768801 PMC2788292

[25] Pathak PP, Pulavarti SVSRK, Jain A, Sahasrabuddhe AA, Gupta CM, Arora A. Solution structure and dynamics of ADF/cofilin from Leishmania donovani. Journal of Structural Biology. 2010;172(3):219–24. 10.1016/j.jsb.2010.07.001 20627129

[26] Singh BK, Sattler JM, Chatterjee M, Huttu J, Schüler H, Kursula I. Crystal Structures Explain Functional Differences in the Two Actin Depolymerization Factors of the Malaria Parasite. Journal of Biological Chemistry. 2011;286(32):28256–64. 10.1074/jbc.M110.211730 21832095 PMC3151070

[27] Yadav R, Pathak PP, Shukla VK, Jain A, Srivastava S, Tripathi S, et al Solution structure and dynamics of ADF from Toxoplasma gondii. Journal of Structural Biology. 2011;176(1):97–111. 10.1016/j.jsb.2011.07.011 21820516 PMC3703439

[28] Dai K, Yuan G, Liao S, Zhang J, Tu X. 1H, 13C and 15N resonance assignments for a putative ADF/Cofilin from Trypanosoma brucei. Biomol NMR Assign. 2011;5(2):249–51. 10.1007/s12104-011-9311-8 21523437

[29] Dong C-H, Tang W-P, Liu J-Y. Arabidopsis AtADF1 is Functionally Affected by Mutations on Actin Binding Sites. Journal of Integrative Plant Biology. 2013;55(3):250–61. 10.1111/jipb.12015 23190411

[30] Dong C-H, Hong Y. Arabidopsis CDPK6 phosphorylates ADF1 at N-terminal serine 6 predominantly. Plant Cell Rep. 2013;32(11):1715–28. 10.1007/s00299-013-1482-6 23903947

[31] Wriggers W, Tang JX, Azuma T, Marks PW, Janmey PA. Cofilin and gelsolin segment-1: molecular dynamics simulation and biochemical analysis predict a similar actin binding mode1. Journal of Molecular Biology. 1998;282(5):921–32. 10.1006/jmbi.1998.2048 9753544

[32] Sept D, Elcock AH, McCammon JA. Computer simulations of actin polymerization can explain the barbed-pointed end asymmetry1. Journal of Molecular Biology. 1999;294(5):1181–9. 10.1006/jmbi.1999.3332 10600376

[33] Pfaendtner J, Cruzb EMDL, Vothc GA. Actin filament remodeling by actin depolymerization factor/cofilin. Proceedings of the National Academy of Sciences. 2010;107(16):7299–304. 10.1073/pnas.0911675107 PMC2867716 20368459

[34] Wong DY, Sept D. The Interaction of Cofilin with the Actin Filament. Journal of Molecular Biology. 2011;413(1):97–105. 10.1016/j.jmb.2011.08.039. 10.1016/j.jmb.2011.08.039 21875597 PMC3184344

[35] Tsui V, Case DA. Theory and applications of the generalized born solvation model in macromolecular simulations. Biopolymers. 2000;56(4):275–91. 10.1002/1097-0282(2000)56:4<275::AID-BIP10024>3.0.CO;2-E 11754341

[36] Kim JI, Kwon J, Baek I, Park HS, Na S. Cofilin reduces the mechanical properties of actin filaments: approach with coarse-grained methods. Physical Chemistry Chemical Physics. 2015;17(12):8148–58. 10.1039/C4CP06100D 25727245

[37] Berman HM, Westbrook J, Feng Z, Gilliland G, Bhat TN, Weissig H, et al The Protein Data Bank. Nucleic Acids Research. 2000;28(1):235–42. 10592235 10.1093/nar/28.1.235 PMC102472

[38] Šali A, Blundell TL. Comparative Protein Modelling by Satisfaction of Spatial Restraints. Journal of Molecular Biology. 1993;234(3):779–815. 10.1006/jmbi.1993.1626 8254673

[39] Lin X, Kaul S, Rounsley S, Shea TP, Benito M-I, Town CD, et al Sequence and analysis of chromosome 2 of the plant Arabidopsis thaliana. Nature. 1999;402(6763):761–8. 10617197 10.1038/45471

[40] Meagher KL, Redman LT, Carlson HA. Development of polyphosphate parameters for use with the AMBER force field. Journal of Computational Chemistry. 2003;24(9):1016–25. 10.1002/jcc.10262 12759902

[41] AMBER parameter database [Internet]. Bryce Group: Computional Biophysics and Drug Design. Available: http://sites.pharmacy.manchester.ac.uk/bryce/amber/.

[42] Hornak V, Abel R, Okur A, Strockbine B, Roitberg A, Simmerling C. Comparison of multiple Amber force fields and development of improved protein backbone parameters. Proteins: Structure, Function, and Bioinformatics. 2006;65(3):712–25. 10.1002/prot.21123 PMC4805110 16981200

[43] Wang J, Wolf RM, Caldwell JW, Kollman PA, Case DA. Development and testing of a general amber force field. Journal of Computational Chemistry. 2004;25(9):1157–74. 10.1002/jcc.20035 15116359

[44] Anandakrishnan R, Aguilar B, Onufriev AV. H++ 3.0: automating pK prediction and the preparation of biomolecular structures for atomistic molecular modeling and simulations. Nucleic Acids Research. 2012;40(W1):W537–W41. 10.1093/nar/gks375 22570416 PMC3394296

[45] Olsson MHM, Søndergaard CR, Rostkowski M, Jensen JH. PROPKA3: Consistent Treatment of Internal and Surface Residues in Empirical pKa Predictions. Journal of Chemical Theory and Computation. 2011;7(2):525–37. 10.1021/ct100578z 26596171

[46] Søndergaard CR, Olsson MHM, Rostkowski M, Jensen JH. Improved Treatment of Ligands and Coupling Effects in Empirical Calculation and Rationalization of pKa Values. Journal of Chemical Theory and Computation. 2011;7(7):2284–95. 10.1021/ct200133y 26606496

[47] Jorgensen WL, Chandrasekhar J, Madura JD, Impey RW, Klein ML. Comparison of simple potential functions for simulating liquid water. The Journal of Chemical Physics. 1983;79(2):926–35. 10.1063/1.445869

[48] Darden T, York D, Pedersen L. Particle mesh Ewald: An N⋅log(N) method for Ewald sums in large systems. The Journal of Chemical Physics. 1993;98(12):10089–92. 10.1063/1.464397

[49] Homeyer N, Gohlke H. Free Energy Calculations by the Molecular Mechanics Poisson−Boltzmann Surface Area Method. Molecular Informatics. 2012;31(2):114–22. 10.1002/minf.201100135 27476956

[50] Kollman PA, Massova I, Reyes C, Kuhn B, Huo S, Chong L, et al Calculating Structures and Free Energies of Complex Molecules: Combining Molecular Mechanics and Continuum Models. Accounts of Chemical Research. 2000;33(12):889–97. 10.1021/ar000033j 11123888

[51] Lee MR, Duan Y, Kollman PA. Use of MM-PB/SA in estimating the free energies of proteins: Application to native, intermediates, and unfolded villin headpiece. Proteins: Structure, Function, and Bioinformatics. 2000;39(4):309–16. 10.1002/(SICI)1097-0134(20000601)39:4<309::AID-PROT40>3.0.CO;2-S 10813813

[52] Weiser J, Shenkin PS, Still WC. Approximate atomic surfaces from linear combinations of pairwise overlaps (LCPO). Journal of Computational Chemistry. 1999;20(2):217–30. 10.1002/(SICI)1096-987X(19990130)20:2<217::AID-JCC4>3.0.CO;2-A

[53] Srinivasan J, Cheatham TE, Cieplak P, Kollman PA, Case DA. Continuum Solvent Studies of the Stability of DNA, RNA, and Phosphoramidate−DNA Helices. Journal of the American Chemical Society. 1998;120(37):9401–9. 10.1021/ja981844

[54] Sitkoff D, Sharp KA, Honig B. Accurate Calculation of Hydration Free Energies Using Macroscopic Solvent Models. The Journal of Physical Chemistry. 1994;98(7):1978–88. 10.1021/j100058a043

[55] Fan J, Saunders MG, Haddadian EJ, Freed KF, De La Cruz EM, Voth GA. Molecular Origins of Cofilin-Linked Changes in Actin Filament Mechanics. Journal of Molecular Biology. 2013;425(7):1225–40. 10.1016/j.jmb.2013.01.020. 10.1016/j.jmb.2013.01.020 23352932 PMC3740545

[56] Pearlman DA, Case DA, Caldwell JW, Ross WS, Cheatham TE Iii, DeBolt S, et al AMBER, a package of computer programs for applying molecular mechanics, normal mode analysis, molecular dynamics and free energy calculations to simulate the structural and energetic properties of molecules. Computer Physics Communications. 1995;91(1–3):1–41. 10.1016/0010-4655(95)00041-D.

[57] Fogolari F, Brigo A, Molinari H. Protocol for MM/PBSA Molecular Dynamics Simulations of Proteins. Biophysical Journal. 2003;85(1):159–66. 10.1016/S0006-3495(03)74462-2 12829472 PMC1303073

[58] Massova I, Kollman PA. Computational Alanine Scanning To Probe Protein−Protein Interactions: A Novel Approach To Evaluate Binding Free Energies. Journal of the American Chemical Society. 1999;121(36):8133–43. 10.1021/ja990935j

[59] Huo S, Massova I Fau—Kollman PA, Kollman PA. Computational alanine scanning of the 1:1 human growth hormone-receptor complex. Journal of Computational Chemistry. 2002;23(1):15–27. 10.1002/jcc.1153 11913381

[60] Ivetac A, McCammon JA. Elucidating the Inhibition Mechanism of HIV-1 Non-Nucleoside Reverse Transcriptase Inhibitors through Multicopy Molecular Dynamics Simulations. Journal of Molecular Biology. 2009;388(3):644–58. 10.1016/j.jmb.2009.03.037 19324058 PMC2744402

[61] Aalten DMFv, Findlay JBC, Amadei A, Berendsen HJC. Essential dynamics of the cellular retinol-binding protein evidence for ligand-induced conformational changes. Protein Engineering. 1995;8(11):1129–35. 10.1093/protein/8.11.1129 8819978

[62] Xue W, Yang Y, Wang X, Liu H, Yao X. Computational Study on the Inhibitor Binding Mode and Allosteric Regulation Mechanism in Hepatitis C Virus NS3/4A Protein. PLoS ONE. 2014;9(2):e87077 10.1371/journal.pone.0087077 24586263 PMC3934852

[63] Gouda H, Kuntz ID, Case DA, Kollman PA. Free energy calculations for theophylline binding to an RNA aptamer: Comparison of MM-PBSA and thermodynamic integration methods. Biopolymers. 2003;68(1):16–34. 10.1002/bip.10270 12579577

[64] Genheden S, Ryde U. Comparison of end-point continuum-solvation methods for the calculation of protein–ligand binding free energies. Proteins: Structure, Function, and Bioinformatics. 2012;80(5):1326–42. 10.1002/prot.24029 22274991

[65] Kato M, Pisliakov AV, Warshel A. The barrier for proton transport in aquaporins as a challenge for electrostatic models: The role of protein relaxation in mutational calculations. Proteins: Structure, Function, and Bioinformatics. 2006;64(4):829–44. 10.1002/prot.21012 16779836

[66] Lappalainen P, Fedorov EV, Fedorov AA, Almo SC, Drubin DG. Essential functions and actin‐binding surfaces of yeast cofilin revealed by systematic mutagenesis. The EMBO Journal. 1997;16(18):5520–30. 10.1093/emboj/16.18.5520 9312011 PMC1170184

