# Supplementary figures and images for: Computational Study of the Binding Mechanism of Actin-Depolymerizing Factor 1 with Actin in Arabidopsis thaliana
Source: PLoS One. 2016 Jul 14;11(7):e0159053. doi: 10.1371/journal.pone.0159053 (PMC4944973; doi:10.1371/journal.pone.0159053)

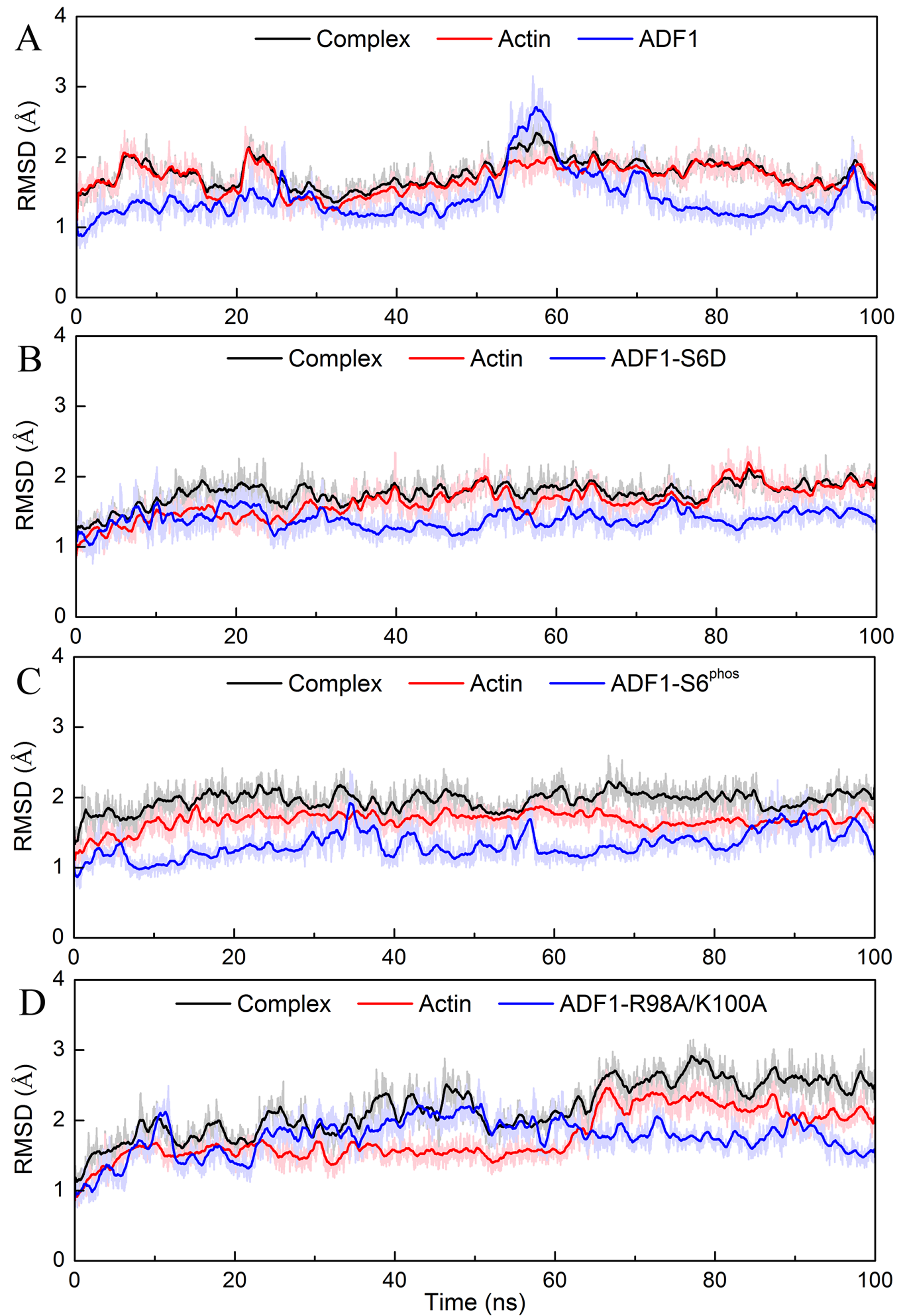

Supplement: S1 Fig — Backbone RMSDs are shown for WT (A), ADF1-S6D (B), ADF1-S6phos (C) and ADF1-R98A/K100A system (D), respectively. (TIF) [file pone.0159053.s001.tif]

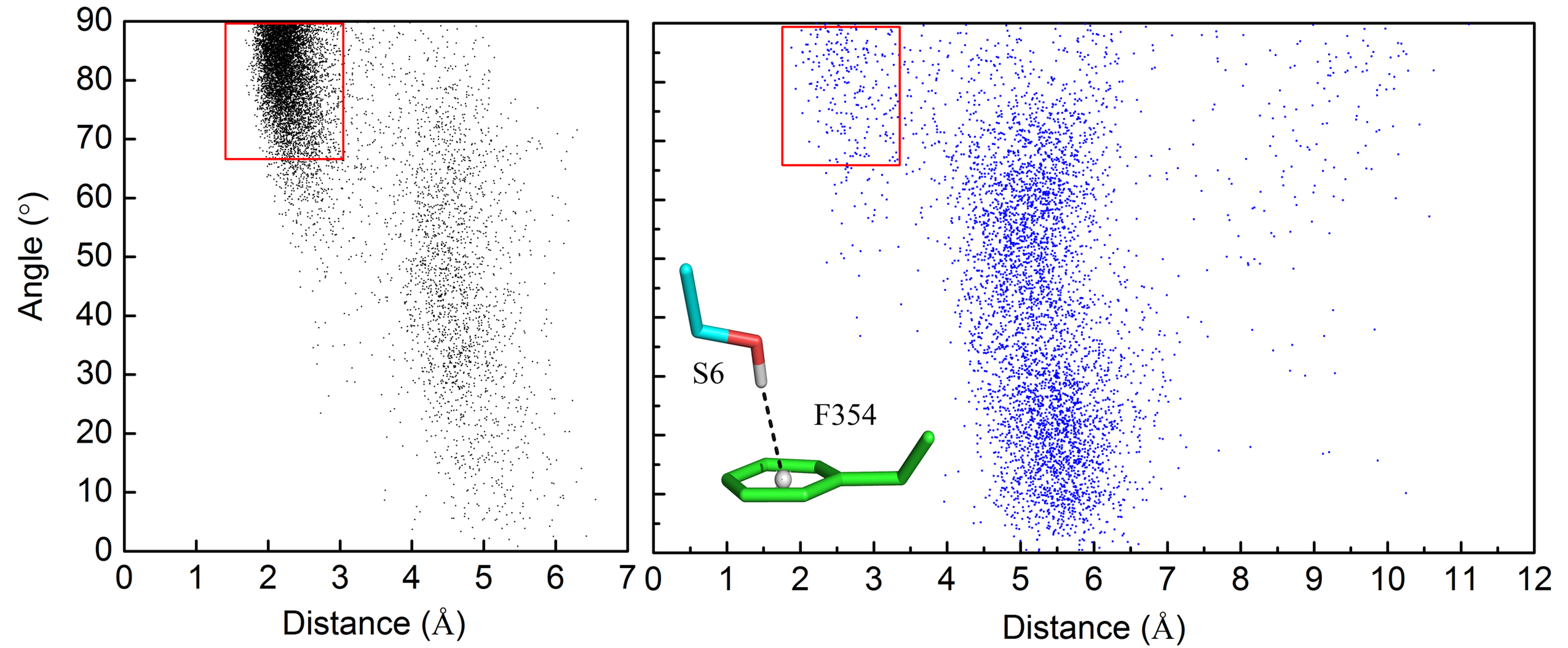

Supplement: S2 Fig — Dots in red square indicate snapshots with strong OH-π interaction. (A). The OH-π interaction of WT system; (B). The OH-π interaction of ADF1-R98A/K100A system. (TIF) [file pone.0159053.s002.tif]
