# Supplementary material for: Computational Study of the Binding Mechanism of Actin-Depolymerizing Factor 1 with Actin in Arabidopsis thaliana
Source: PLoS One. 2016 Jul 14;11(7):e0159053. doi: 10.1371/journal.pone.0159053 (PMC4944973; doi:10.1371/journal.pone.0159053)
Supplement: S1 Table — (DOCX) [file pone.0159053.s003.docx]

Table S1 Binding free energy for the WT and mutated systems according to the MM-GB/PBSA methods based on different protein dielectric constants (kcal/mol).

| **ε** | Method |  |  | MM-GB/SA | | | MM-PB/SA | | |
| --- | --- | --- | --- | --- | --- | --- | --- | --- | --- |
|  | System | $\Delta E_{\text{ele}}$ | $\Delta E_{\text{vdw}}$ | $\Delta G_{\text{SA}}$ | $\Delta G_{\text{GB}}$ | $\Delta G_{\text{total}}$ | $\Delta G_{\text{SA}}$ | $\Delta G_{\text{PB}}$ | $\Delta G_{\text{total}}$ |
| 1 | WT | -572.37±2.70 | -107.15±0.24 | -14.29±0.03 | 604.56±2.56 | -89.26±0.37 | -13.81±0.03 | 620.33±2.59 | -73.00±0.45 |
|  | S6D | -583.14±2.40 | -111.78±0.27 | -15.45±0.04 | 621.69±2.22 | -88.69±0.37 | -14.90±0.02 | 639.21±2.33 | -70.62±0.48 |
|  | R98A/K100A | -465.62±2.45 | -107.49±0.29 | -14.98±0.04 | 501.71±2.31 | -86.38±0.44 | -14.98±0.04 | 522.63±2.42 | -65.46±0.51 |
| 2 | WT | -286.19±1.35 | -107.15±0.24 | -14.29±0.03 | 298.45±1.26 | -109.18±0.28 | -13.81±0.03 | 304.76±1.28 | -102.38±0.28 |
|  | S6D | -291.57±1.20 | -111.78±0.27 | -15.45±0.04 | 306.91±1.10 | -111.89±0.31 | -14.89±0.03 | 313.88±1.28 | -102.88±0.30 |
|  | R98A/K100A | -232.81±1.22 | -107.49±0.29 | -14.98±0.04 | 247.68±1.14 | -107.60±0.34 | -14.98±0.03 | 257.59±1.19 | -97.69±0.32 |
| 4 | WT | -143.09±0.67 | -107.15±0.24 | -14.29±0.03 | 145.40±0.62 | -119.14±0.26 | -13.81±0.03 | 148.05±0.60 | -116.01±0.25 |
|  | S6D | -145.79±0.60 | -111.78±0.27 | -15.45±0.04 | 149.52±0.53 | -123.50±0.29 | -14.90±0.02 | 152.38  ±0.56 | -119.64±0.25 |
|  | R98A/K100A | -116.40±0.61 | -107.49±0.30 | -14.98±0.03 | 120.66±0.56 | -118.21±0.32 | -14.98±0.04 | 124.52±0.58 | -114.36±0.29 |
