# Supplementary material for: Computational Study of the Binding Mechanism of Actin-Depolymerizing Factor 1 with Actin in Arabidopsis thaliana
Source: PLoS One. 2016 Jul 14;11(7):e0159053. doi: 10.1371/journal.pone.0159053 (PMC4944973; doi:10.1371/journal.pone.0159053)
Supplement: S2 Table — (DOCX) [file pone.0159053.s004.docx]

Table S2 The contributions of the important residues for the binding of ADF1 with actin (kcal/mol)

| Residue | Interaction energy | | | |
| --- | --- | --- | --- | --- |
|  | ADF1-WT | ADF1-S6D | ADF1-S6^phos^ | ADF1-R98A/K100A |
| Asp13 | -0.33±0.01 | -1.36±0.02 | -0.16±0.01 | 0.64±0.01 |
| Asp 26 | -0.39±0.01 | -3.4±0.13 | -0.14±0.00 | -0.04±0.00 |
| Glu109 | -0.85±0.02 | -0.92±0.01 | -1.20±0.01 | -0.58±0.001 |
| Tyr135 | -0.25±0.02 | 0.19±0.03 | -2.23±0.04 | -1.61±0.06 |
| Leu142 | -0.59±0.02 | -0.50±0.02 | -0.50±0.01 | -1.25±0.02 |
| Tyr145 | -4.24±0.03 | -3.47±0.05 | -3.58±0.04 | -2.67±0.03 |
| Ala146 | -1.71±0.03 | -2.22±0.02 | -1.99±0.02 | -0.66±0.02 |
| Ser147 | 0.07±0.02 | -1.10±0.03 | -0.14±0.02 | -0.94±0.02 |
| Gly148 | -1.89±0.04 | -1.72±0.04 | -1.23±0.04 | -1.56±0.04 |
| Arg149 | -10.34±0.15 | 0.90±0.08 | -1.29±0.09 | 1.22±0.11 |
| Thr150 | -2.52±0.07 | -2.91±0.05 | -2.91±0.07 | -2.47±0.08 |
| Asp156 | -0.16±0.01 | -1.15±0.02 | -0.04±0.01 | -0.27±0.01 |
| Glu169 | 5.36±0.12 | 5.82±0.12 | 9.95±0.16 | 8.93±0.17 |
| Asp290 | 0.15±0.01 | -0.03±0.01 | 3.26±0.14 | 3.38±0.10 |
| Asp294 | 2.88±0.11 | 0.90±0.08 | 3.19±0.14 | 7.35±0.16 |
| Asn298 | -0.22±0.01 | -0.34±0.01 | -2.20±0.04 | -0.91±0.02 |
| Lys328 | -0.77±0.01 | -2.73±0.12 | -1.21±0.06 | -2.93±0.12 |
| Lys330 | -1.32±0.09 | -6.16±0.16 | -2.78±0.12 | -2.12±0.10 |
| Pro335 | -0.64±0.01 | -0.18±0.00 | -0.27±0.01 | -1.57±0.02 |
| Glu336 | -0.55±0.03 | 3.32±0.18 | -0.44±0.05 | 0.01±0.03 |
| Arg337 | -0.27±0.00 | 1.05±0.03 | -0.23±0.01 | -0.79±0.01 |
| Ser340 | -0.37±0.02 | -0.16±0.03 | -0.44±0.01 | -1.04±0.02 |
| Ile343 | -2.11±0.02 | -2.87±0.03 | -0.98±0.01 | -1.01±0.01 |
| Gly344 | -0.50±0.01 | -1.01±0.02 | -0.04±0.02 | -0.18±0.02 |
| Ile347 | -3.55±0.02 | -3.48±0.03 | -3.71±0.02 | -2.98±0.02 |
| Leu348 | -0.84±0.01 | -1.10±0.02 | -1.12±0.01 | -1.99±0.02 |
| Leu351 | -2.26±0.02 | -1.56±0.03 | -2.62±0.02 | -2.63±0.02 |
| Thr353 | -4.53±0.06 | -4.84±0.05 | -3.55±0.05 | -2.52±0.07 |
| Phe354 | -4.60±0.05 | -4.19±0.03 | -4.32±0.02 | -2.32±0.03 |
| Gln355 | -0.32±0.01 | -0.29±0.01 | -4.49±0.06 | -0.14±0.00 |
| Gln356 | -3.51±0.09 | -2.13±0.06 | -0.68±0.02 | 0.16±0.01 |
| Met357 | -0.83±0.02 | -2.34±0.04 | -1.67±0.03 | -0.76±0.02 |
| Lys375 | 1.84±0.09 | 0.10±0.02 | 0.13±0.03 | -1.40±0.07 |
| Phe377 | -0.69±0.02 | 2.9±0.11 | -0.67±0.01 | 0.74±0.05 |
| ADF1 | | | | |
| Ala2 | 0.41±0.08 | -4.58±0.21 | 2.11±0.08 | -2.32±0.10 |
| Asn3 | -0.03±0.05 | -0.76±0.05 | -3.75±0.04 | -0.22±0.02 |
| Ala4 | -2.57±0.04 | -2.97±0.04 | -1.94±0.02 | -2.02±0.02 |
| Ala5 | -2.35±0.04 | -1.93±0.04 | -1.73±0.02 | -1.67±0.02 |
| Ser6 | -2.43±0.03 | 1.17±0.08 | 6.17±0.17 | 0.43±0.08 |
| Glu36 | 1.30±0.07 | 1.29±0.06 | 1.29±0.06 | 1.06±0.05 |
| Lys39 | -1.02±0.04 | -1.18±0.07 | -1.45±0.04 | -1.16±0.04 |
| Lys84 | -0.73±0.00 | -0.69±0.00 | -0.86±0.01 | -1.04±0.01 |
| Asp93 | 1.19±0.06 | 4.65±0.13 | 2.21±0.08 | 1.51±0.07 |
| Lys96 | -1.94±0.06 | -1.77±0.05 | -1.46±0.01 | -1.42±0.01 |
| Val97 | -4.64±0.02 | -4.41±0.03 | -4.51±0.02 | -5.77±0.03 |
| Arg98 | -1.84±0.05 | -5.77±0.13 | -2.25±0.04 | -1.36±0.02 |
| Lys100 | -1.09±0.06 | 3.42±0.13 | -2.21±0.06 | -1.98±0.02 |
| Met101 | -5.02±0.03 | -5.94±0.04 | -6.43±0.03 | -6.15±0.03 |
| Ile102 | -1.89±0.02 | -2.36±0.02 | -1.54±0.02 | -2.30±0.02 |
| Ala104 | -2.39±0.02 | -2.21±0.02 | -2.47±0.02 | -2.96±0.02 |
| Ser 105 | -1.50±0.02 | -1.32±0.02 | -1.54±0.02 | -0.95±0.02 |
| Lys107 | -8.02±0.09 | -7.35±0.13 | -5.77±0.27 | 2.58±0.30 |
| Asp108 | -0.85±0.03 | 1.59±0.06 | 4.11±0.15 | 3.41±0.09 |
| Lys111 | -1.42±0.02 | -1.51±0.04 | -5.30±0.20 | -6.43±0.17 |
| Arg112 | -0.75±0.00 | -1.09±0.01 | -1.78±0.04 | -1.45±0.02 |
| Glu120 | 1.48±0.03 | 1.88±0.05 | 2.50±0.07 | 2.28±0.13 |
| Gln122 | -1.41±0.06 | -0.54±0.02 | -0.92±0.05 | -4.02±0.08 |
| Thr124 | -4.90±0.07 | -1.18±0.07 | -2.38±0.06 | -1.07±0.06 |
| Glu128 | 5.15±0.10 | 4.45±0.10 | 4.23±0.17 | 3.14±0.11 |
| Asp132 | 0.34±0.01 | 0.38±0.00 | 1.56±0.07 | 1.93±0.07 |
| Arg137 | -1.10±0.11 | 0.65±0.02 | -7.06±0.27 | -11.61±0.14 |
